# To the Editors of the Medical and Physical Journal

**Published:** 1803-08-01

**Authors:** 


### *To the Editors of the Medical and Physical Journal.*

GENTLEMEN,

IN your 52d Number for January 1803, Mr. Low has stated a method by which metallic casts may be obtained from the osseous cavities of the human ear. The same process has been described in the Lectures on the Art of making Anatomical Preparations, as delivered by Dr. Baillie and the late Mr. Cruikshank, full ten years since, and that process was regularly mentioned for at least five successive years, to the writer's knowledge, as well as the person's name who contrived it. Other casts, of a similar kind, had been occasionally made, but the certainty of success was fixed by the method of coating the temporal bone, and heating it so as to expel the contained air. The compound fusible metal is liable to oxydation at the red heat which is necessary to be given to the bone, so that grain tin is found to make a more beautiful cast. A tube must also be fixed into the meatus externus, in order that a greater pressure of the melted metal should urge its flow into the small cavities. A coating of pipe clay is better than one of Paris plaster; but if the latter be used, it should be altogether included in a crucible, to prevent cracks and flaws, which will often drain out the metal before it sets, and spoil the cast.

Preparations of this kind have been long used at the lectures in Windmill-street, and those were presented to the teacher by Mr. Carlisle, who contrived the method of making them. I am, yours, &c.

REMEMBRANCER.

---

